# Activating the healing process: three-dimensional culture of stem cells in Matrigel for tissue repair

**DOI:** 10.1186/s12896-024-00862-5

**Published:** 2024-05-25

**Authors:** Shukui Xu, Liru Zhao, Yinghui Li, Xiuge Gu, Ziyang Liu, Xing Han, Wenwen Li, Wensheng Ma

**Affiliations:** 1 Department of Orthodontics, Hebei Key Laboratory of Stomatology, Hebei Clinical Research Center for Oral Diseases, School and Hospital of Stomatology, Hebei Medical University, Shijiahzuang, 050017 China; 2 Department of Orthodontics, School of Medicine, Tianjin Stomatological Hospital, Nankai University, Tianjin, 300041 China; 3 Tianjin Key Laboratory of Oral and Maxillofacial Function Reconstruction, Tianjin, 300041 China

**Keywords:** Gingival mesenchymal stem cells, Three-dimensional culture, Matrigel matrix, Tissue engineering, Soft tissue repair

## Abstract

**Background:**

To establish a strategy for stem cell-related tissue regeneration therapy, human gingival mesenchymal stem cells (hGMSCs) were loaded with three-dimensional (3D) bioengineered Matrigel matrix scaffolds in high-cell density microtissues to promote local tissue restoration.

**Methods:**

The biological performance and stemness of hGMSCs under 3D culture conditions were investigated by viability and multidirectional differentiation analyses. A Sprague‒Dawley (SD) rat full-thickness buccal mucosa wound model was established, and hGMSCs/Matrigel were injected into the submucosa of the wound. Autologous stem cell proliferation and wound repair in local tissue were assessed by histomorphometry and immunohistochemical staining.

**Results:**

Three-dimensional suspension culture can provide a more natural environment for extensions and contacts between hGMSCs, and the viability and adipogenic differentiation capacity of hGMSCs were significantly enhanced. An animal study showed that hGMSCs/Matrigel significantly accelerated soft tissue repair by promoting autologous stem cell proliferation and enhancing the generation of collagen fibers in local tissue.

**Conclusion:**

Three-dimensional cell culture with hydrogel scaffolds, such as Matrigel, can effectively improve the biological function and maintain the stemness of stem cells. The therapeutic efficacy of hGMSCs/Matrigel was confirmed, as these cells could effectively stimulate soft tissue repair to promote the healing process by activating the host microenvironment and autologous stem cells.

**Graphical abstract:**

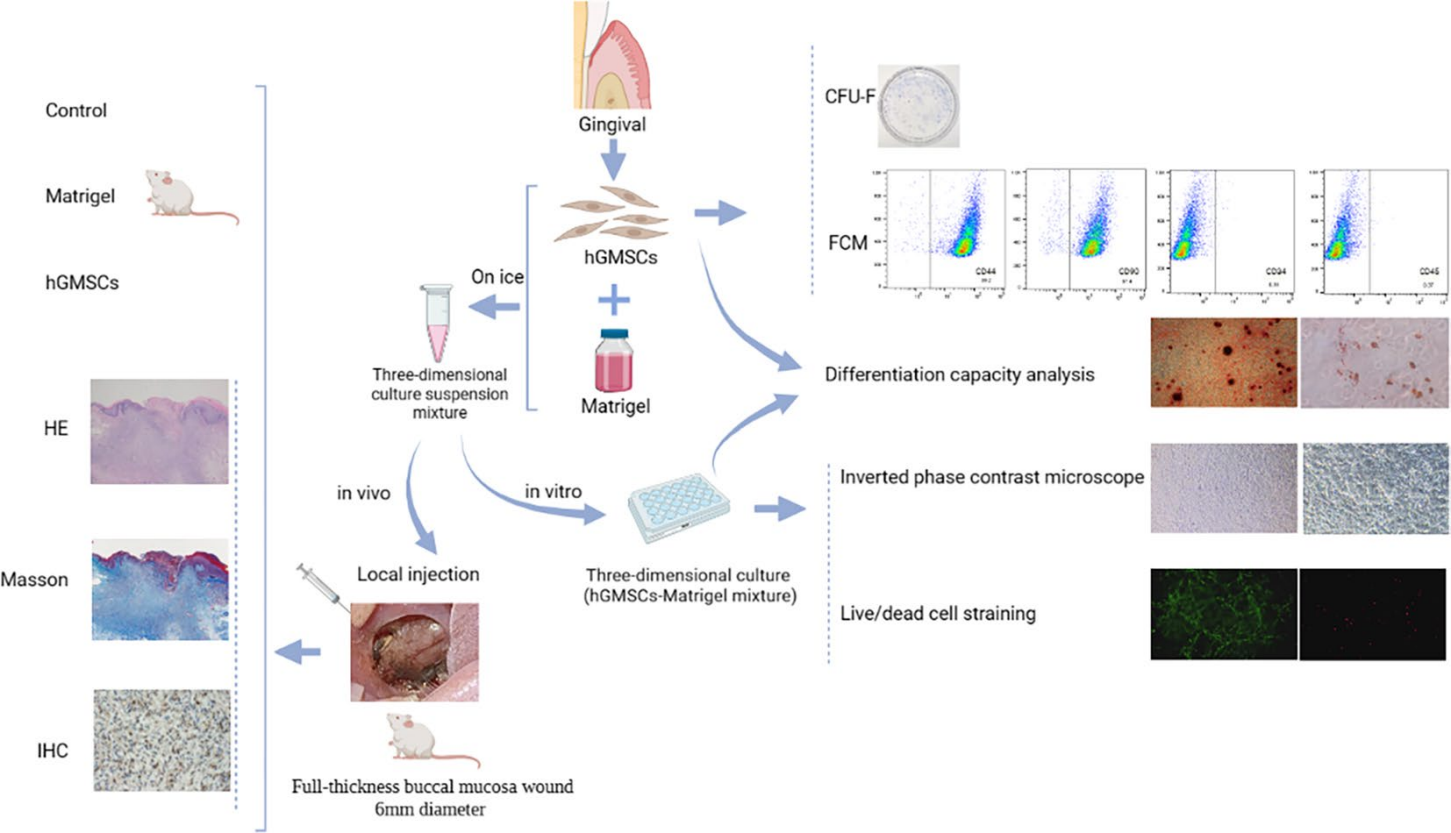

## Background

Oral soft tissue defects are frequently caused by infection, immune-related disease, iatrogenic practices or chronic mechanical irritation [1]. In particular, major aphthous ulcers are defined as deep tissue defects that can penetrate even beyond the muscle layers, causing severe pain and delaying healing for weeks or months without targeted treatment [2]. In previous studies, stem cell-related tissue regeneration therapy has been considered a promising approach for treating deep soft tissue defects [3, 4]. Several studies have indicated the potential advantages of human dental-derived mesenchymal stem cell (MSC)-based approaches for tissue engineering [5, 6]. Among all dental-derived MSCs, human gingival mesenchymal stem cells (hGMSCs) have a stronger ability to resist infection and disease after transplantation and have been demonstrated to exhibit strong immunoregulatory functions [4, 7]. Therefore, hGMSCs have been used as selectable seed cells for soft tissue regenerative therapies and relevant stem cell-based biology.

To improve the properties of hGMSCs for tissue engineering, three-dimensional (3D) culture in the context of bioengineered scaffolds could be considered [8]. As a biological and resorbable mixture of extracellular matrix (ECM) proteins and growth factors (GFs), the Matrigel matrix is widely used as a basement membrane matrix to support the growth and function of target cells in cell engineering applications [9]. Because of its self-healing properties, which are based on temperature, Matrigel is suitable as a scaffold for loading hGMSCs into high-cell density microtissues when its mechanical properties are restored [10].

The animals selected for oral soft tissue defect models mainly include rabbits, rats and guinea pigs. As a common experimental animal, the Sprague‒Dawley (SD) rat has received widespread attention and is commonly used because of its economy, ease of operation, and simplicity of the modeling process. The objective of this study was to establish a stem cell-related tissue regeneration therapy strategy in which hGMSCs were loaded with 3D bioengineered Matrigel matrix scaffolds into high-cell density microtissues to promote local tissue restoration, which was stronger than the effect of injecting adherent hGMSCs or Matrigel alone.

## Materials and methods

### Isolation and culture of cells

hGMSCs were obtained from gingival tissues around the extracted third molars, which were extracted due to orthodontic needs or impaction. The patients did not have diabetes or infectious diseases. The study complied with the Declaration of Helsinki, and informed consent forms were obtained from all subjects or from a legal representative. For this experiment, gingival tissues from 10 healthy donors were used, and the tissue size was approximately 5 × 5 × 5 mm.

The epithelial layer of gingival tissue was removed manually using a sterile surgical blade and washed with phosphate-buffered saline (PBS) (BioInd, Kibbutz Beit Haemek, Israel) supplemented with penicillin/streptomycin (PS) (BioInd, Kibbutz Beit Haemek, Israel) (PBS-PS; PBS supplemented with 5% PS). The samples were visually assessed under a stereomicroscope (Olympus, Tokyo, Japan). This step was repeated until the epithelial layer was completely removed. Before being transferred to a 25 cm^2^ culture flask (BioInd, Kibbutz Beit Haemek, Israel), the tissues were minced into pieces < 3 mm in diameter and then cultured with primary culture medium (α­minimum essential medium (α­MEM) (Gibco, New York, USA) containing 20% fetal bovine serum (FBS) (Gibco, New York, USA) and 1% PS). The pieces were cultured in a humidified incubator at 37 °C with 5% CO_2_ until the cells had grown. After 1–2 weeks of primary culture, residual epithelial cells were removed using a cell scraper under a microscope, and adherent stem cells were harvested with 0.25% trypsin-EDTA (1X) (Gibco, New York, USA). Subculture was performed to ensure the purity of the stem cells using growth medium (α-MEM supplemented with 15% FBS). The growth medium was changed every 3 days until the cells reached 80–90% confluence, at which point the cells were passaged regularly. Cells were used between the second and fifth passages. Stem cells from each subject were identified and used separately to avoid mixing. Stem cells from the same subject were used in the same batch of experiments to avoid within-group bias due to individual differences in stem cell viability.

### Phenotype analysis by flow cytometry

Surface antigens of hGMSCs were detected using flow cytometry analysis at the second passage (P2). Following digestion, adherent cells were harvested by centrifugation at 15,000 rpm for 10 min at 4 °C and washed with PBS. The suspended cells were incubated with monoclonal antibodies conjugated with fluorescent dyes (Abcam, Cambridge, UK) in the dark at 4 °C for 40 min (for each sample, the initial dilution was 1:10 and the final dilution was 1:50). Antigen–antibody complexes were detected within two hours using a flow cytometer. The following antibodies were used: a CD44 antibody, a CD90 antibody and a negative control (CD34, CD45, and anti-human IgG).

### Colony-forming unit-fibroblast assay

To assess the colony-forming efficiency of hGMSCs, 1 × 10^3^ single cells in suspension (P2) were plated in a 10 cm culture dish in growth medium (α-MEM supplemented with 20% FBS) and cultured in a humidified incubator at 37 °C with 5% CO_2_. After 10 days, the cells were fixed in 4% paraformaldehyde (Solarbio, Beijing, China) for 30 min, stained with 1% toluidine blue (Solarbio, Beijing, China) for 15 min, washed with distilled water and dried for evaluation. Aggregates of ≥ 50 cells were scored as colonies, and total colony numbers were then counted under an inverted phase contrast microscope (Olympus, Tokyo, Japan), while aggregates of < 50 cells or faintly stained cells were excluded. The colony-forming unit–fibroblast ratio (CFU-F) was subsequently calculated as follows: CFU-F (%) = total number of colonies/1000 × 100%. The culture was repeated three times. The bottom plate was divided into 25 squares for cell counting, and each square was marked with visible lines.

### Three-dimensional cell culture

The biocompatibility of the 3D scaffolds and their stem cell-loading ability were confirmed by 3D cell culture in vitro. The 3D scaffolds for stem cells were formed with a Matrigel matrix (Corning, Corning NY, USA) (#354,234, protein concentration of 8.9 mg/mL). The Matrigel matrix was thawed slowly at 4 °C overnight, and hGMSCs solution was added to the 3D scaffolds so that the cells could be absorbed into the 3D scaffolds. hGMSCs suspensions (1 × 10^6^ cells in 150 µL of PBS) were mixed well with an equal volume of Matrigel matrix (150 µL) with a pipetting device on ice. The hGMSCs/Matrigel mixture was spread evenly in each well of a 24-well plate (250 µL of mixture per well, cell density of 3333 cells/µL) and plated in an incubator (37 °C) for 30 min to allow the Matrigel to solidify. After growth medium (α-MEM with 20% FBS) was added (500 µL per well), the mixture was cultured in an incubator (37 °C with 5% CO_2_) according to standard protocols. On days 0, 1, 3, 5 and 8, the 3D distribution of the cells and the soma extension of the cells were observed with an inverted phase contrast microscope (Olympus, Tokyo, Japan).

### Cell viability analysis

The cell viability of the hGMSCs/Matrigel mixture was assessed with a live/dead staining kit (Solarbio, Beijing, China). The experiments were performed according to the kit instructions. After seeding, the mixture was stained on days 1, 3, 5, and 8, followed by submersion in staining solution for 40 min in the dark. Cell viability was visualized via inverted fluorescence microscopy (Olympus, Tokyo, Japan). Viable cells were marked with Calcein-AM (Ex/Em = 490/515 nm), whereas dead cells were marked with propidium iodide (PI) (Ex/Em = 535/617 nm). The cells were counted under a microscope at 200× magnification (*n* = 6 per group). Three fields of view were randomly selected for each well of the 24-well plate, and the data were averaged. The dead cell ratio (DCR) was calculated as follows: DCR = the number of dead cells/the total number of cells×100%.

### Differentiation capacity analysis

The hGMSCs/Matrigel mixture was loaded into a 24-well plate (250 µL of mixture per well). When the cells reached 100% confluence and stopped proliferating, osteogenic medium containing 5 mmol/L β-glycerophosphate sodium (Solarbio, Beijing, China), 50 mg/L ascorbic acid (Solarbio, Beijing, China) and 100 nmol/L dexamethasone (Solarbio, Beijing, China) was added. The osteogenic medium was refreshed every 3 days. After 31 days of osteogenic induction, the cultures were stained with Alizarin Red S (Beyotime, Shanghai, China) according to the kit instructions. The control group (hGMSCs subjected to adherent culture) was induced and strained in the same way and at all the same time points as above. The percentage of the Alizarin Red-positive area was analyzed using Image-Pro Plus at 100× magnification.

Adipogenic differentiation was induced for the hGMSCs/Matrigel mixture and the control group (hGMSCs subjected to adherent culture) using an hMSC Adipogenic Differentiation Medium Kit (Oricell, Guangzhou, China) when the cells reached 100% confluence and stopped proliferating. After 31 days of adipogenic induction, Oil Red O staining (Solarbio, Beijing, China) was performed to detect lipid droplets. The percentage of Oil Red O-positive area was analyzed using Image-Pro Plus under 200× magnification. Each differentiation experiment was repeated at least three times. Three fields of view were randomly selected for each well of the 24-well plate, and the data were averaged.

### Rat soft tissue defect model and treatment

**Fig. 1:**
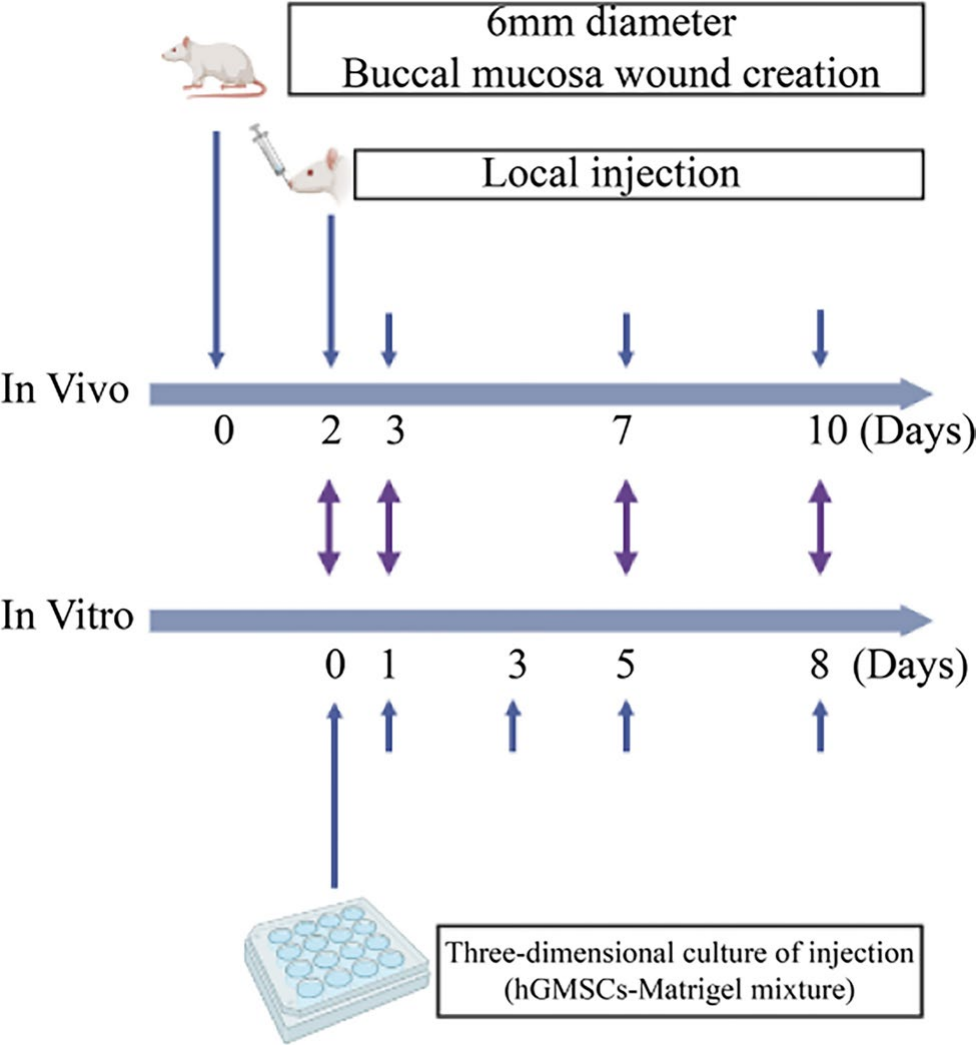
Experimental design and timeline for the in vivo and in vitro experiments in rats

The in vitro experiments needed to be followed up with in vivo experiments. Figure 1 shows the experimental approach. The experiments included 72 male Sprague–Dawley (SD) rats, aged 7–8 weeks and weighing 250–260 g. Rats were purchased from Beijing Weitong Lihua Biotechnology Co., Ltd. (Beijing, China). The experimental rats were randomly allocated into four equal groups (18 rats each): the experimental group (hGMSCs/Matrigel group) and three control groups. Then the four groups were randomly stratified according to their respective time sampling points on days 3, 7, and 10 post-modeling (*n* = 6). Each cage contained 3 rats in a standard animal room (24 ± 1 °C, 50 ± 10% humidity, natural lighting conditions). Adequate food and drinking water were provided and litter was changed in the cage every 2 days.

Each cage contained animals from a single group. All rats were allowed to adapt for 1 week before surgery. The rats were anesthetized by intraperitoneal injection of sodium pentobarbital (60 mg/kg). After the rats were subjected to deep anesthesia, a full-thickness buccal mucosa wound was created using a 6 mm diameter dermatological punch, which was chemical corrosion with formocresol (Longly, Wuhan, China) for 1 min. The wounds were graded by measuring the wound depth with a sterile blunt probe and photographed on day 0. The surgical procedures were standardized and performed by the same operator to minimize the variability of the model. For all animal surgeries, postoperative analgesia and a soft diet were provided. All rats were carefully observed after the operation for their status, food intake and drinking water. Body weight was measured daily. Body weight change = the rats’ body weight from the current day − body weight from the previous day.

On day 2, the wounds had formed scabs and the material for local injection was prepared. The hGMSCs/Matrigel mixture (300 µL per rat) was injected into the center and surroundings of the wound area at a depth below the submucosa according to the clinical experience of the operation and the recording of the wound depth described above in the experimental group. The injection speed was closely controlled to avoid spillage, and the cells were observed for 30 min after injection to allow the Matrigel to solidify. Successful injection was defined as no obvious spillage of the injected material when the wound was lightly touched with sterile tweezers. The other three groups were the Matrigel group (150 µL of Matrigel with 150 µL of PBS), the hGMSCs group (1 × 10^6^ cells in 300 µL of PBS), and the control group (healing process without any interventions).

### Histological analysis

To probe the healing process of local tissue macroscopically, rats in each group were photographed after anesthesia under a stereomicroscope (Olympus, Tokyo, Japan), and then the rats were euthanized using the CO_2_ euthanasia method according to their respective time sampling points. Wound size (WS) was calculated (using ImageJ software, version 1.53, *n* = 6 per group).

The full-thickness buccal mucosa and the muscular layer were retrieved aseptically and fixed in 4% paraformaldehyde for 24 h. After fixation, the specimens were placed in biopsy cassettes (Citotest, Jiangsu, China), dehydrated with graded ethanol, processed in a tissue-processing machine (Leica, Weztlar, Germany), and embedded in paraffin blocks. Sections with a thickness of 4 μm were cut. A portion of each section was stained using a hematoxylin-eosin (HE) staining kit (Baso, Zhuhai, China) according to the manufacturer’s instructions.

A Masson’s trichrome staining kit (Leagene, Beijing, China) was used according to the kit instructions to determine the degree of collagen maturity. Individual pictures (40×) of Masson staining were stitched and converted into a panoramic picture. The percentage of collagen area in each panoramic image (*n* = 6 per group) was calculated with ImageJ as follows: percentage of collagen area = area of collagen fibers/area of the epithelial layer ×100%. Data analysis was performed at least three times by blinded evaluators and the results from different evaluators were then averaged.

### Immunohistochemical analysis

Proliferating cell nuclear antigen (PCNA) was detected via immunohistochemical (IHC) staining. Sections were dewaxed in water and placed in citric acid antigen repair solution (Boster, Wuhan, China) for heat repair. After blocking endogenous peroxidase with 3% hydrogen peroxide for 20 min and washing with PBS, the sections were blocked with 5% goat serum (Boster, Wuhan, China) for 30 min and incubated with primary antibody (rabbit anti-rat PCNA antibody; the dilution used for PCNA was 1:200; Immunogen Range:201–261/261) (Bioss, Beijing, China) at 4 °C overnight and with secondary antibody (goat anti-rabbit) (ZSGB-BIO, Beijing, China) at RT for 30 min. Staining was visualized using diaminobenzidine (DAB). All stained sections were imaged under a motorized fluorescence microscope (BX63, Olympus, Tokyo, Japan). To calculate the number of PCNA + cells, the mean optical density (MOD) was used to denote the staining intensity, and data from a minimum of three randomly selected microscopic visual fields per section were averaged and are presented as the mean ± standard deviation (SD) using Image-Pro Plus software, version 6.0.0.260. MOD = total optical density/the area of positive cells. Data analysis was performed at least three times by blinded evaluators, and the results from different evaluators were then averaged.

### Statistical analysis

All experiments were performed at least three times, and the data are expressed as the mean ± standard deviation (mean ± SD). One-way ANOVA was used to analyze the significance of differences between groups. The statistical analyses were conducted with SPSS (version 21.0), and differences at *P* < 0.05 were considered to indicate statistical significance. Figures were prepared using GraphPad Prism software (version 8.0.1), Biorender (webstie: https://www.biorender.com, Toronto, Canada) and Adobe Illustrator (version 27.0). * indicates *P* < 0.05, ** indicates *P* < 0.01, *** indicates *P* < 0.001.

## Results

### Isolation and characterization of hGMSCs

**Fig. 2:**
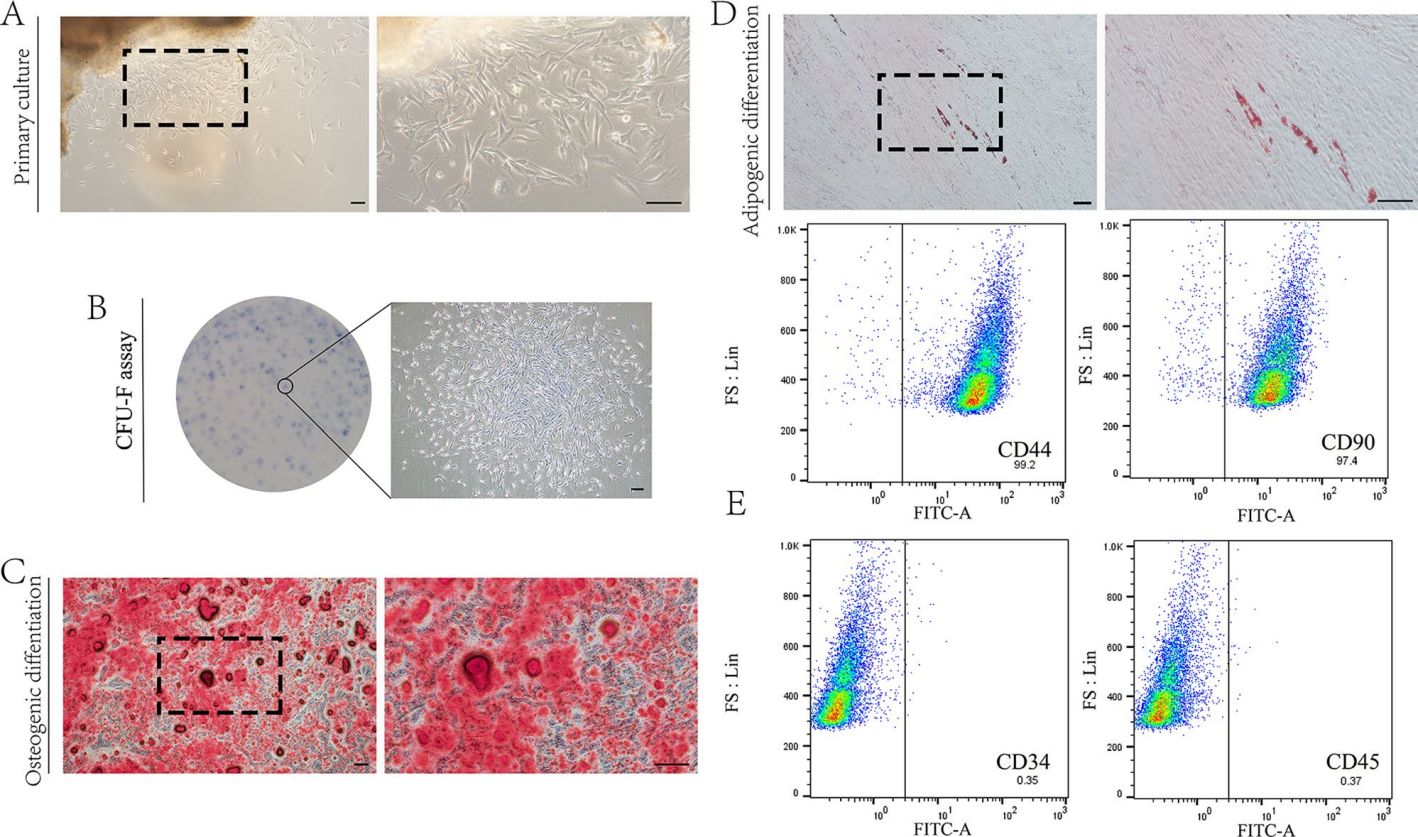
Culture and identification of hGMSCs. (**A**) Representative images of primary cultures of hGMSCs are shown on the left (40×), and the corresponding enlarged images of the left are shown on the right (100×). (**B**) Representative images of colony-forming unit–fibroblast assays of hGMSCs. A typical colony is shown on the right (40×), and the corresponding visual inspection image of the left are shown on the left. (**C**) Representative images of Osteogenic differentiation of hGMSCs are shown on the left (40×) and right (100×). Each scale bar (**A**–**C**) is 200 μm. (**D**) Representative images of the adipogenic differentiation of hGMSCs are shown on the left (100×) and right (200×), and each scale bar is 100 μm. (**E**) Representative phenotype analysis results obtained by flow cytometry. MSCs surface markers (CD44 and CD90) are shown in the upper insets, and hematopoietic cell markers (CD34 and CD45) are included below

**Fig. 3:**
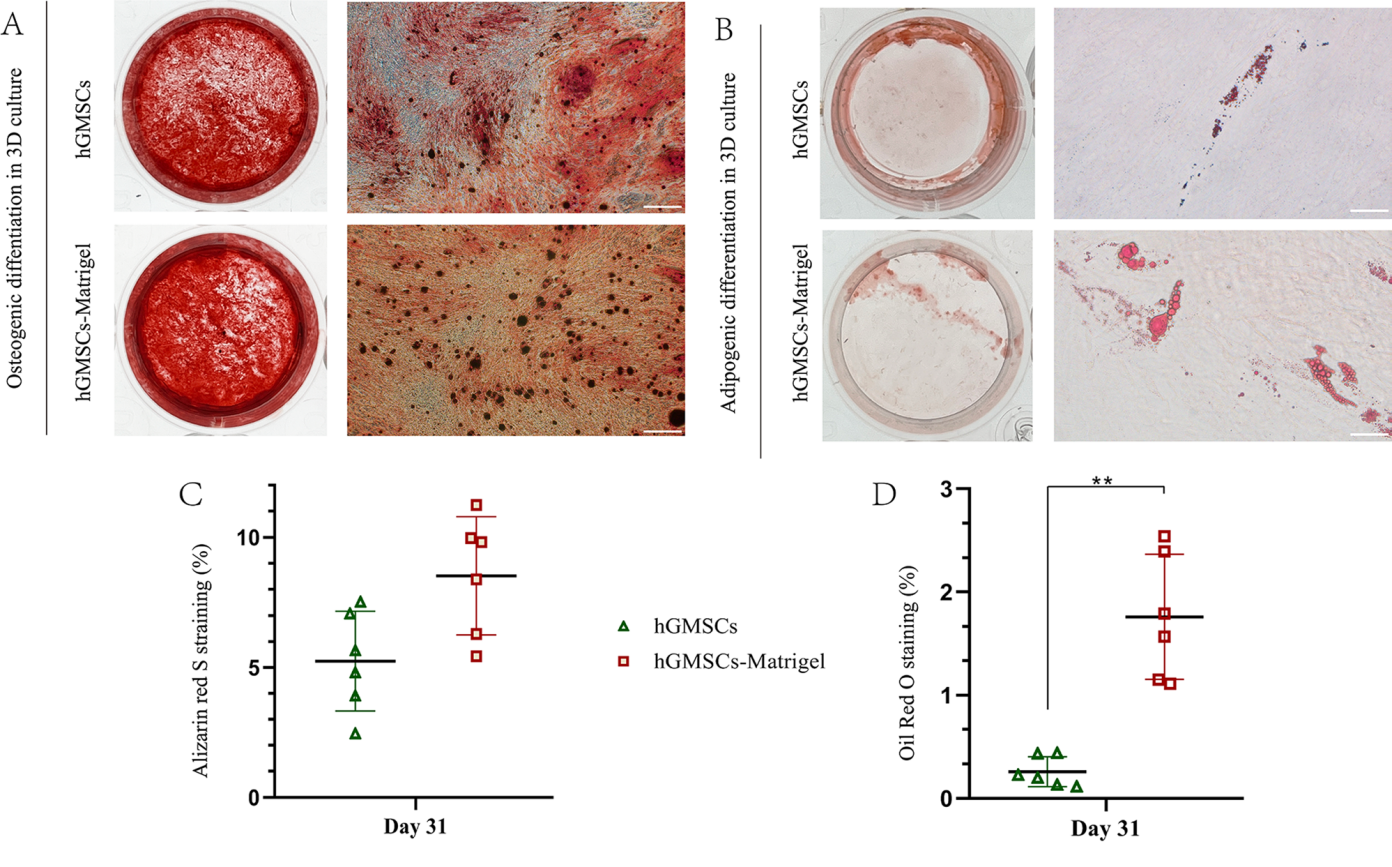
Differentiation capacity analysis. (**A**) Representative images of the osteogenic differentiation of hGMSCs and hGMSCs/Matrigel. Each scale bar is 200 μm, and the magnification is 40×. (**B**) Representative images of adipogenic differentiation of hGMSCs and hGMSCs-Matrigel. Each scale bar is 50 μm, and the magnification is 400×. (**C**) Analysis of Alizarin Red S staining. (**D**) Analysis of Oil Red O staining. * indicates *P* < 0.05, ** indicates *P* < 0.01. All experiments were performed at least three times, and one-way ANOVA was used to analyze the significance of differences between groups

hGMSCs were successfully isolated from gingival tissues using a standard tissue block culture method (Fig. 2A). The morphotype of the primary cells was fusiform or polygonal. After passaging, the cells became more uniform, had a fibroblast-like morphology and had stable proliferation rates. The percentage of CFU-F was 40.87% ± 3.09% after culturing for 10 days, which indicated that the cells were able to proliferate well (Fig. 2B). Osteogenic and adipogenic differentiation abilities were determined after 31 days of culture in differentiation induction medium (Fig. 2C–D). Flow cytometry data showed that the tested cells expressed MSC surface markers (CD44 and CD90) and were negative for hematopoietic cell markers (CD34 and CD45) (Fig. 2E). Thirty-one days after osteogenic and adipogenic induction, Alizarin Red-positive nodules and Oil Red O-positive lipid droplets were observed in the hGMSCs (Fig. 3A–B), which confirmed the differentiation capacity of the isolated cells.

### Three-dimensional culture enhanced the viability and differentiation capacity of hGMSCs

**Fig. 4:**
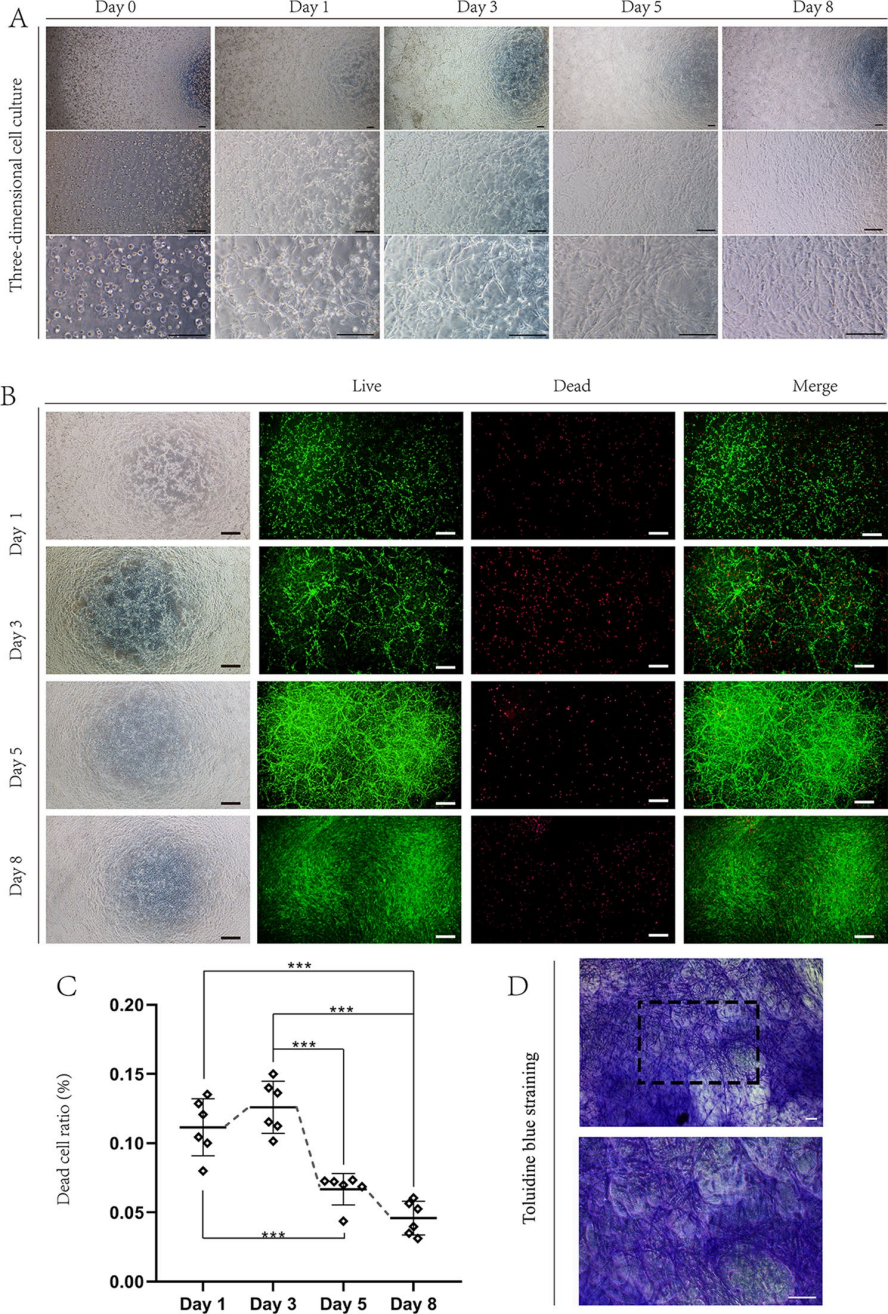
Three-dimensional cell culture. (**A**) Representative images of 3D cell culture with inverted phase contrast microscopy. Each scale bar is 200 μm, and the magnifications from top to bottom are 40×, 100× and 200×. (**B**) Representative images of live/dead-stained 3D cell cultures obtained via inverted fluorescence microscopy (40×). Each scale bar represents 500 μm. (**C**) Statistical analysis of the number of dead cells. * indicates *P* < 0.05, ** indicates *P* < 0.01, *** indicates *P* < 0.001. (**D**) Representative images of toluidine blue-stained of hGMSCs in 3D culture on day 10. Each scale bar in C is 100 μm and the magnifications are 100× (top) and 200× (bottom). All experiments were performed at least three times, and one-way ANOVA was used to analyze the significance of differences between groups

To probe the effects of the biomaterial on hGMSCs survival and performance, in vitro culture was performed, and the cells were observed under an inverted phase contrast microscope (Fig. 4A). The suspended cells were sufficiently incorporated into the Matrigel at multiple levels of 3D culture, and the cells rapidly adapted to the in vivo-like environment in which the soma fully extended and contacted other cells in different 3D layers to achieve stereo network formation in the scaffold within one day. The surviving cells in hGMSCs/Matrigel in 24-well plates could be classified according to two different regions: (1) cells close to the bottom of the plate still formed adherens junctions and proliferated rapidly to increase the interaction area; and (2) cells embedded in the Matrigel scaffold maintained multiangle intercellular communication (Fig. 4B–D). The significant decrease in the dead cell ratio between day 3 and day 5 was due to increased numbers of new cells (Fig. 4C).

The adipogenic differentiation capacity of hGMSCs under 3D culture conditions was significantly enhanced. Lipid droplet staining by Oil Red O was significantly greater in 3D culture than in adherent hGMSCs on day 31 (Fig. 3B–D), but no significant increase in the area of calcified nodules stained with Alizarin Red S was found compared to that in adherent hGMSCs on day 31 (Fig. 3A–C).

### hGMSCs/Matrigel accelerated local tissue restoration in vivo

**Fig. 5:**
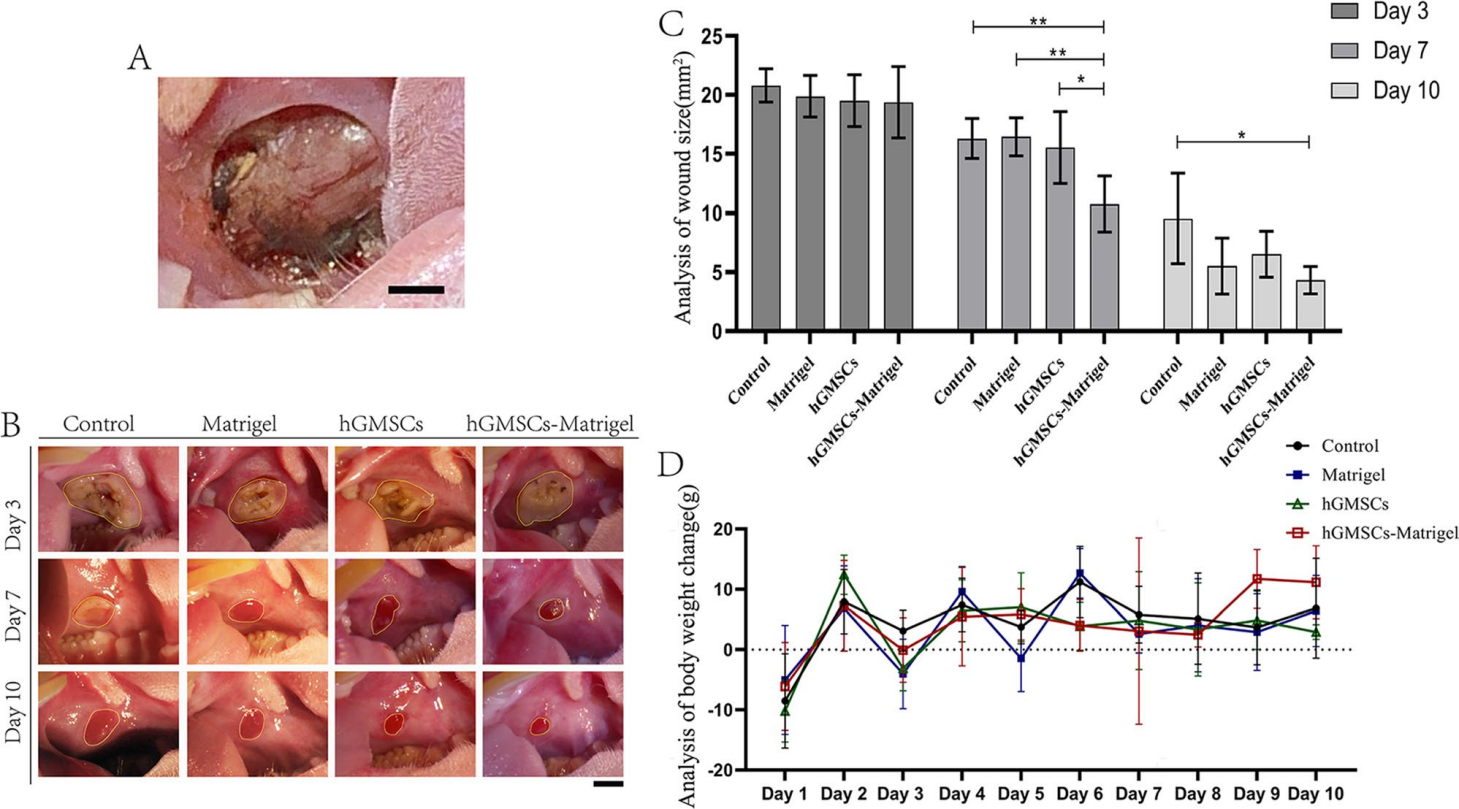
Wound model and treatment. (**A**) Representative images of full-thickness buccal mucosa wounds on day 0 under visual inspection. (**B**) Representative images of wound size changes on days 3, 7, and 10 under visual inspection. The areas of the wounds are circled in yellow. Each scale bar (**A**, **B**) is 2 mm. (**C**) Statistical analysis of wound size.* indicates *P* < 0.05, ** indicates *P* < 0.01. (**D**) Analysis of body weight change. The mean and standard deviation (SD) of the changes are shown in the figure. All experiments were performed at least three times, and one-way ANOVA was used to analyze the significance of differences between groups

Full-thickness buccal mucosa wounds 6 mm in diameter were created (Fig. 5A). Quantitative analysis of wound size revealed a significant decrease after hGMSCs/Matrigel transplantation compared to those of the other groups, especially on day 7 (Fig. 5B–C). In addition, the control group without any interventions exhibited soft tissue defects with significantly delayed concrescence, and the efficacy of hGMSCs/Matrigel persisted. The weight changes revealed no significant differences between groups at any time point (Fig. 5D), indicating that the treatments did not influence the status of the rats.

**Fig. 6:**
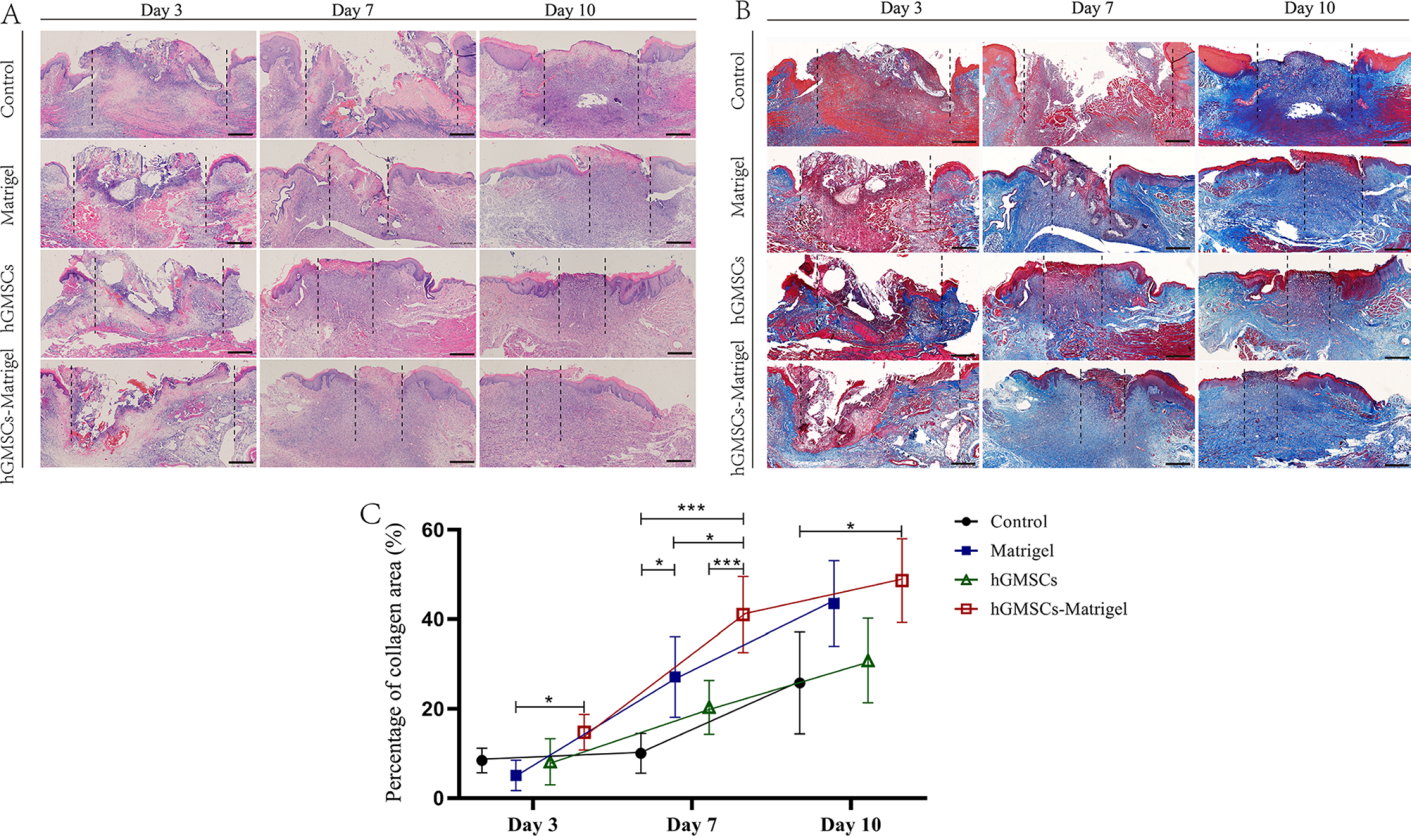
Histological analysis. (**A**) Representative images of hematoxylin-eosin staining. (**B**) Representative images of Masson’s trichrome staining. Each scale bar (**A**, **B**) is 500 μm. Individual images (40×) were stitched and converted into a panoramic images in A and B. (**C**) Statistical analysis of the percentage of collagen area. * indicates *P* < 0.05, ** indicates *P* < 0.01. All experiments were performed at least three times, and one-way ANOVA was used to analyze the significance of differences between groups

HE staining and Masson’s trichrome staining were used to evaluate therapeutic potential in terms of histological features at the largest diameter of the wound (Fig. 6A–B). Increased generation of new collagen fibers indicating a greater reduction in epithelial barrier defects was observed on days 7 and 10 after hGMSCs/Matrigel injection (Fig. 6C). Notably, the degree of collagen maturity also improved to some extent on day 7 in the group injected with Matrigel only and could be either associated with or independent of the contents of ECM proteins and GFs.

**Fig. 7:**
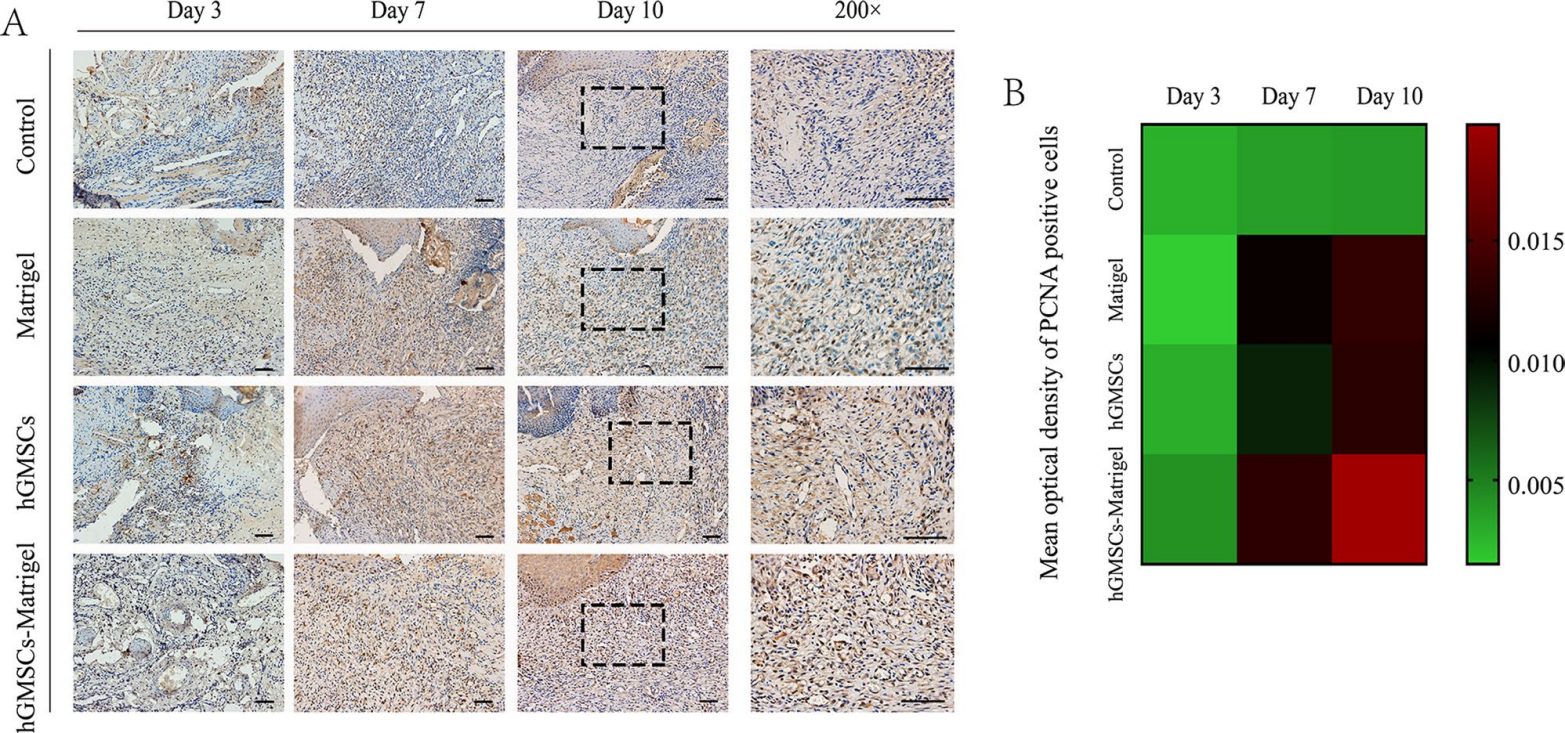
Immunohistochemical analysis. (**A**) Representative images of IHC staining of PCNA. Each scale bar is 200 μm. The magnification is 100× on days 3, 7, and 10, and the corresponding enlarged images of day 10 are shown on the right (200×). (**B**) Numerical properties of the MOD of PCNA + cells are visualized as a heatmap. The grids of the heatmap use a color scale to display a color mapped from its numeric value, in which a trend toward ‘red’ indicates ‘a large amount’ and a trend toward ‘green’ indicates ‘not at all or very little’ of PCNA + cells

Local endogenous stem cell proliferation at the target site was visualized via PCNA protein expression (Fig. 7A–B), and cells proliferated more rapidly toward the wound center on days 7 and 10 after hGMSCs/Matrigel treatment. There were no significant differences between the groups on day 3 (one day after transplantation), indicating that the therapeutic efficacy of hGMSCs/Matrigel requires time to accumulate.

## Discussion

Severe soft tissue defects are often difficult to heal due to their large area and depth, resulting in healing with scarring or adhesion formation [11–14]. The currently available treatments aim to accelerate soft tissue defect healing and restore the histological characteristics and physiological functions of the oral mucosa as much as possible [2]. Previous studies have noted that the wound healing process is complex and involves a set of genetically programmed repair processes [15–17], and therapeutic validation of human MSCs may be divided into two major aspects [18, 19]. First, to verify their ectopic regeneration capacity, exogenous MSCs may undergo proliferation and differentiation and ultimately replace damaged tissues and restore their function, such as those in xenografts in immunodeficient animal models or autologous transplantation [20, 21]. Second, exogenous MSCs may exert immunoregulatory effects via the release of growth factors, the awakening of tissue progenitor cells for regeneration, and the remodeling of the ECM and tissue structure in a process called cell empowerment [10, 22]. Studies have indicated that local transplantation of GMSCs may help accelerate the healing process of wounds through inhibition of the local inflammatory response, proliferation of autologous stem cells and neovascularization [2, 12]. GMSCs exhibit the features and functions of mesenchymal stem cells [23]; in particular, GMSCs exhibit inflammation-related changes in terms of differentiation and proliferation [4, 7], and GMSCs are more accessible in discarded biological samples in the clinic. Therefore, local GMSCs transplantation offers opportunities to accelerate the regeneration of damaged mucosal soft tissue to its original form and function.

The underlying mechanisms that explain the protective effect of MSCs remain to be elucidated. Recent studies have shown that the actual therapeutic mechanisms of MSCs transplantation are not the result of the proliferation and direct replacement of allogeneic donor cells. Instead, the therapeutic effect may result from the modulation of the host microenvironment and activation of autologous stem cells [18, 24]. Immune rejection is a key issue of stem cell transplantation [25] in either allografts or xenografts. The interaction between exogenous MSCs and endogenous immune cells is an important link in the activation of endogenous stem cell-mediated repair processes, including intervention in the immune response induced by exogenous MSCs and immunogenic cell death mediated by the immune response [18, 26–28]. Some studies have demonstrated that the immunomodulatory function of MSCs requires inflammatory responses for their activation. Although steroid-based anti-inflammatory therapy (such as dexamethasone) is often used to reduce severe inflammatory responses, such as graft-versus-host disease (GvHD), the results of these studies show that the concomitant application of MSCs with steroids may interfere with MSCs efficacy [29, 30]. Under these premises, stem cell regulation to promote local tissue restoration is mediated by the coordinated interplay between cellular networks, called the stem cell niche, which refers to an in vivo or in vitro stem cell microenvironment that interacts with stem cells to regulate cell fate and is composed of allogeneic stem cells, ECM, autologous stem cells and other factors [31–33]. In the stem cell niche, exogenous MSCs can secrete and recruit a variety of bioactive molecules after transplantation [34]. Among these molecules, interleukin-1 receptor antagonist (IL-1RA), via the Fas/Fap-1/Cav-1 cascade, TGF-β and IL-10 were reported to activate endogenous stem cells to promote both self-renewal and differentiation for ECM remodeling and to inhibit the excessive inflammatory response, which helps accelerate wound healing [16, 35–38]. Our study showed that after hGMSCs transplantation, the microenvironment of the wound was regulated, with anti-inflammatory effects during the wound healing process. PCNA + cells were more numerous and generated more collagen fibers postwounding, indicating that the host microenvironment and autologous stem cells were activated and that the repair process was accelerated.

Various 3D cellular culture methods have been reported and have provided new insight into improving hMSC properties for tissue engineering [8, 39]. Hydrogels are often designed to provide an ideal physicochemical environment for the growth of cells in tissue engineering [40]. In our study, the 3D culture conditions used a commercial matrix (Matrigel) as a self-supporting gel scaffold, and the cells were deposited into the supporting scaffold, which was subsequently fused to 3D high-cell density microtissues [40]. 3D suspension culture can provide a more natural environment for extensions and contacts between cells, and significant differences in the morphology, cytoskeletal structure and intercellular adhesive forces between cells were found in 3D culture compared to those in adherent culture, which could be intrinsic factors influencing the properties of MSCs [41, 42]. Studies have shown that 3D cell culture can effectively improve the biological function and maintain the stemness of stem cells by improving their anti-inflammatory activities and multidirectional differentiation ability via the activation of Wnt signaling [43] and altering their paracrine functions via suppression of the NF-kB pathway but activation of the PI3K/AKT pathway in cranial stem cells [44]. In addition, hydrogel scaffolds combined with ECM in the niche could form a barrier for allogeneic donor cells to reduce the killing efficiency of local inflammation and autoimmunity and to prolong the residence time of encapsulated MSCs [40, 45, 46]. Furthermore, a study indicated that 3D conditions can improve the resistance of stem cells to local oxidative stress-induced apoptosis by increasing the contact area between cells and tissue fluid [47], which increases endogenous cell proliferation and promotes angiogenesis at the same time [48]. In our study, increases in viability and differentiation capacity were exhibited by hGMSCs after 3D culture preconditioning in vitro, and better treatment efficacy in terms of soft tissue regeneration and wound closure than local hGMSCs injection alone was observed, indicatings that pretreatment with 3D encapsulate can optimize the therapeutic potential of stem cell therapies to a certain extent.

More effective routes of administration, such as hyaluronic acid adhesive microneedle patches [49] and in vitro-engineered mucosa equivalents [12], have also been investigated in recent studies for promoting oral mucosal regeneration. These routes of administration allow for early restoration of coherence at the sites of tissue defects compared with syringes. Rodents are often chosen from an economic perspective. However, the oral vestibule of rodents is shallow, and the buccal mucosa is relatively small. Moreover, the thickness of the buccal mucosa and submucosa is thin and loose compared with that of the human oral mucosa, and the vascular supply is not as abundant as that of the human oral mucosa [50]. Therefore, the experimental effect will be limited by the routes of administration and the histological characteristics of the rodents; thus, there is still a long way to go before these findings can be used in the clinic. The major limitation of our study is that we explored tissue healing from a histological and local cellular perspective only and did not further explore the underlying mechanisms involved. Future studies should be performed to investigate the detailed mechanisms involving exogenous MSCs and endogenous immune cells in a trauma setting.

## Conclusion

In this study, 3D cell cultures formed by hydrogel scaffolds, such as Matrigel, effectively improved the biological function and maintained the stemness of stem cells, including growth status, viability and adipogenic differentiation capacity. The effect of local transplantation of hGMSCs/Matrigel was evaluated in a full-thickness buccal mucosa wound model. The therapeutic efficacy of hGMSCs/Matrigel was confirmed, as these cells could effectively stimulate soft tissue repair to promote the healing process by activating the host microenvironment and autologous stem cells. Thus, this study provides a promising therapeutic strategy for soft tissue regeneration that combines stem cells and ECM scaffolds.

## Data Availability

No datasets were generated or analysed during the current study.
